# Determinants of Incomplete Childhood Vaccination among Children Aged 12–23 Months in Gambela Region, Southwest Ethiopia: A Case Control Study

**DOI:** 10.4314/ejhs.v31i1.8

**Published:** 2021-01

**Authors:** Asnake Mebrat, Lamessa Dube, Ayantu Kebede, Zemedu Aweke

**Affiliations:** 1 Gambela Health Office, Gambela, Ethiopia; 2 School of public health, Jimma University, Jimma, Ethiopia; 3 Department of Anesthesia, Dilla University, Dilla, Ethiopia

**Keywords:** Determinants, vaccination, case control, Gambela, Ethiopia

## Abstract

**Background:**

Childhood vaccination is considered as one of the most cost-effective public health interventions. With an increasing dropout rate from vaccination, the factors for incomplete vaccination are not well explored. The objective of this study was to identify determinants of incomplete childhood vaccination.

**Method:**

Community based case-control study was conducted from March 1–30, 2018. Cases were children who missed at least one dose of routine vaccine while controls were children who completed all recommended doses. Face-to-face interviews were used to collect data. Multivariable logistic regression was performed in order to identify determinants with 95% CI and a p-value of <0.05.

**Result:**

A total of 93 cases and 185 controls were participated in the study. Not attending postnatal care [AOR=2.16, 95% CI: 1.08–4.28], household not visited by health workers [AOR=3.99, 95% CI: 2.13–7.48], postponing vaccination schedules [AOR = 6.15, 95% CI: 3.08–12.27], caretakers who had misconception of vaccination [AOR = 2.90, 95% CI: 1.53–5.52], unsatisfied care takers [AOR=1.970, 95% CI:1.04–3.74] and poor knowledge about vaccines [AOR = 2.33, 95% CI: 1.19–4.59] were determinants of incomplete childhood vaccination.

**Conclusion:**

Failure to attend postnatal care, postponing vaccination schedules, having misconception for vaccine contraindication, households not visited by health workers, caretakers who had poor knowledge about vaccines and unsatisfied caretakers were determinants of incomplete childhood vaccination. Based on the finding, it is recommended that health education should be improved to decrease caretakers' misconception, poor knowledge and postponement of the vaccine schedule. It is also recommended to increase health workers household visit.

## Introduction

Childhood vaccination is one of the most cost effective public health interventions to avert child morbidity, mortality and disability (1). However, high vaccine uptake rates, specific to each Vaccine-Preventable Diseases (VPD) are needed for community-level immunity to be achieved and sustained so that lowering the disease risk beyond what would be predicted by coverage of vaccine alone (2,3). Ethiopia strictly follows WHO recommendation schedules for developing countries to benefit the full potential of immunization. Currently, routine vaccines administered are BCG and OPV at birth; OPV, Pentavalent vaccine (DTP), Hib conjugate and hepatitis B virus vaccine), and Pneumococcal Conjugate Vaccine (PCV) at ages 6, 10 and 14 weeks; Rota Virus Vaccine (RV) at 6 and 10 weeks; and Measles Containing Vaccine (MCV) at nine months (4,5).

Although coverage of individual vaccine is improved in Ethiopia, the proportion of completely vaccinated children with all recommended vaccines is rarely achieved (6). Children who were not vaccinated or incompletely vaccinated to all recommended vaccines are at greater risk for contracting VPD and transmitting diseases to children too young to be vaccinated, people with vaccine contraindication and people with vaccine failure (7,8).

Globally, about 20.8 million children in the same age group had failed to receive a single dose of MCV. Around 60% of these children live in 10 countries, including Ethiopia. Among 19.5 million infants, 12.8 million (66%) did not receive the first DTP dose, and 6.6 million (34%) started but did not complete the DTP series (9,10).

Based on the EDHS of 2016, only four in ten children aged 12–23 months (39%) received all basic vaccinations at some time, and 22% received these vaccinations before their first birthday (11). According to the Health Information Management System report, Fully Immunized Children (FIC) under one year of age had also reached 82.9% in 2006 (9). However, a survey done in selected zones of Ethiopia in 2015 showed that the overall FIC among children aged 12–23 months was 69% (12).

According to the administrative immunization coverage report in Gambela Regional Health Bureau (GRHB), FIC was 68% in 2017 with a dropout rate of 11.8% from penta1 to 3, and 21.45% from penta1 to measles. EDHS of 2016 also showed FIC was only 41% in the Gambela region(11,13). The coverage of complete vaccination in Ethiopia is low (11, 12). Some pieces of literature which were done in this area are mainly focused on the factors affecting the complete vaccination. With an increasing vaccine dropout rate in the country and on the study area, exploring the determinants for incomplete vaccination is still needed. Therefore, this study aimed to identify determinants of incomplete vaccination among children aged 12–23 months living in Lare district, Gambela region.

## Methods and Materials

**Study area and design**: A community based unmatched case-control study was conducted in the Lare district, the Nuer zone of Gambela region, south-west Ethiopia from May 1–30, 2018. Lare district is one of the five districts in Nuer zone of Gambela regional government. It is located 846km away from Addis Ababa and 80km from Gambela town, the capital city of Gambela regional state. It has a population of 45801, of which 6183 are under-five and 1374 are under one year children. There are two health centers and 10 health posts, of these four health posts, were functional for EPI service in the district.

**Source population and study population**: The source population was all children aged 12–23 months who had at least started one immunization program in the study area. Cases were children aged 12–23 months who had missed at least one dose of the recommended vaccine as identified from immunization registry book. Controls were children aged 12–23 months who had completed the entire recommended vaccine, identified from immunization registry book.

**Sample size determination and sampling procedure**: The sample size was calculated by stat calc epi info version 7 using determinant variables from a previous case-control study done in Ethiopia (14). The predictor variable, no PNC followup, was selected since it gives maximum sample size. Among controls and cases, 66.4% and 83.2% of mothers had no PNC followup, respectively. By using an assumption of 95% CI, 80% power, 10% non-response rate, and the control-to-case ratio of 2:1, the total sample size was 293 (98 cases and 195 controls). The inclusion criteria were children who had a full address from EPI registry book and caretakers of children living in the study area at the time of the study. The exclusion criteria were caretakers who changed their residence or if the child was dead. All eligible cases and controls were extracted from EPI registration book of 4 health posts and 2 health centers. After extraction, sampling frames were prepared for cases and controls separately. Finally, 98 cases and 195 controls were selected by simple random sampling method using computer-generated random numbers. After extracting eligible cases and controls, study participants were contacted a week before the actual data collection by using the name of ‘kebele’ and ‘gote’ as well as the name of the child and name of mothers or caretakers. Both cases and controls were extracted from EPI registry book.

**Data collection**: Four diploma-holder nurses were trained for data collection, and two BSc-holder nurses were recruited for supervision. Training was given for data collectors and supervisors on how to facilitate the data collection to assure data quality. Both data collectors and supervisors were selected from the health centers. The data were collected through face-to-face interviews with the mothers/caregivers and a review of the immunization cards. A structured questionnaire adapted from WHO 2015 cluster survey manual, Ethiopia EPI survey and literature were used. The questionnaire was prepared in English and translated into the local language Nuer and then back-translated into English to ensure consistency. The Nuer version of the questionnaire was used for data collection. The questionnaire included sociodemographic variables of mothers/caretakers and children, factors related to mothers/caretakers, service utilization related factors and provider-related factors. A pre-test was made on 5% of the sample size at health centers other than the study area, and the necessary correction was made thereafter. The supervisors supervised the face-to-face interviews and also checked the likeness of actual cases with that from the registry.

**Data processing and analysis**: The data was entered into Epi-data version 3.1 and exported to SPSS 20 for analysis. Frequency tables and descriptive summaries were used to describe the study variables by univariate analysis. Bivariate logistic regression analysis was used to see the significance of the association between dependent and independent variables. All explanatory variables which had an association in the bivariate analysis at p-value less than or equal to 0.25 were entered into multivariable logistic regression model by backwards elimination method. Multi-collinearity between significant variables in bivariate analysis with a p-value less than 0.25 was checked by variance inflation factor before entered to a multivariable regression. Adjusted odds ratio with their 95% CI was used to identify the determinants of incomplete vaccination.

**The following operational definitions are used.**

**Knowledge about schedules of vaccination**: Mothers were asked four questions which are related to the schedule of vaccination which includes age at which the child begins vaccination, how many times a child should visit vaccination site to be fully vaccinated, at what age the child should complete immunization and how to know whether a child complete vaccination or not. The right answers were given a value of 1, and for incorrect answers a value of 0 was given. After computing the sum for each respondent, it was dichotomized into having good knowledge about schedules if the sum of the value was greater than 2, and having poor knowledge about schedules if the sum of the value was less than or equal to 2.

**Knowledge about vaccination**: Caretakers were asked questions related to the type of vaccine given, for which diseases the vaccine was given. The care taker was categorized as having good level of knowledge if she/he could mention at least three vaccines either by their name or by their route (site) of administration or which diseases they were meant for.

**Knowledge about the benefit of vaccination**: If mothers/caretakers know and could mention at least one type of benefit of vaccination, the mothers/caretakers were categorized as having good knowledge about benefit of vaccination.

**Caretakers' satisfaction towards service provider**: Mothers/caretakers were asked ten satisfaction related questions using a Likert scale (strongly disagree to strongly agree) which has five options. Each variable was measured on 5 points starting from 1 to 5. The mean score was then computed. The score was dichotomized into satisfied if it is greater than or equal to the mean score and unsatisfied if the mothers/caretakers scored less than the mean score.

**Misconception for vaccine contraindication**: Mothers/caretakers were asked six questions using Likert scale. Each variable was measured on 5 points starting from 1 for strongly disagree to 5 for strongly agree. The mean score was computed and dichotomized into no misconception if the score was less than or equal to the mean and has misconception if the score was greater than the mean score.

**Ethical consideration**: Ethical clearance was obtained from Jimma University Research Ethics Review Committee. A formal letter was submitted to GRHB and Lare district by Jimma University. Permission was obtained from GRHB and Lare district. Written informed consent was obtained from each study participant. Study participants' right to withdraw from the study at any points was respected. The confidentiality was maintained by avoiding personal identifiers and using code.

## Results

A total of 278 (93 cases and 185 controls) were included in this study, making a response rate of 94.8%. The mean age of caretakers among cases was 26.17(SD ±5.556) years, and it was 27.7 (SD±6.580) years among controls. Caretakers´ marital status, ethnicity and religion were married, 88(94.6%), Nuer, 88(94.6%), and protestants, 72(77.4%), among cases, and among controls, 183(98.9%) were married, 178(96.2%) were Nuer and 150(81.1%) protestants, respectively. The median birth interval of children under study among cases and controls was 26(SD±4.9) and 27(SD±5.3) months, respectively. The remaining sociodemographic characteristics are shown in (Table 1).

**Table 1:** Sociodemographic characteristics of care takers and children under study in Lare district, Nuer Zone, Gambela Region, 2018

Variables	Categories	Cases N (%)	Controls N (%)
Age of care taker	<27	43(46.2)	106(57.3)
	≥27	50(53.8)	79(42.7)
Educational status of care	No formal education	37(39.8)	42(22.7)
taker	Primary (1–8)school	28(30.1)	81(43.8)
	Secondary and above	28(30.1)	62(33.5)
Marital status	Married	88(94.6)	183(98.9)
	Others	5(5.4)	2(1.1)
Ethnicity	Nuer	88(94.6)	178(96.2)
	Others^1^	5(5.4)	7(3.8)
Residence	Urban	20(21.5)	48(25.9)
	Rural	73(78.5)	137(74.1))
Mobility of care takers	Yes	28(30.1)	42(22.7)
	No	65(69.9)	143(77.3)
Religion	Protestant	72(77.4)	150(81.1)
	Catholic	11(11.8)	25(13.5)
	Others^2^	10(10.8)	10(5.4)
Family size	≤4	32(34.4)	77(41.6)
	>4	61(65.6)	108(58.4)
Occupation of care taker	Farmer	27(29)	51(27.6)
	Merchant	22(23.7)	36(19.5)
	Employer	22(23.7)	39(21.1)
	Daily labourer	3(3.2)	6(3.1)
	House wife	19(20.4)	53(28.6)
Child birth order	1	15(16.1)	38(20.5)
	2–4	49(52.7)	113(61.1)
	≥5	29(31.2)	34(18.4)
Sex of child	Male	43(46.2)	97(52.4)
	Female	50(53.8)	88(47.6)
Birth interval	<=24	41(44.1)	52(28.1)
(in months)	25–36	48(51.6)	125(67.6)
	>=37	4(4.3	8(4.3)

Caretakers' utilization of health service-related factors showed that 63 (67.7%) of mothers among cases and 144 (77.8 %) of mothers among controls attended ANC at least once during their pregnancy. Among these mothers who had ANC checkup, only 19 (30.2%) of cases and 74 (51.4%) of controls were attended the third and fourth round. After delivery of the child, 23 (24.7%) of mothers among cases and 100 (54.1%) of caretakers among controls attended PNC at least once. With regards to accessibility of vaccination site for caretakers, 61.3% from cases and 59.5% from controls spent less than 30 minutes to reach the vaccination site. With regards to missed opportunity, 56 (60.2%) of cases and 76 (41.1%) controls experienced returning without getting vaccines for their eligible child (Table 2). Regarding caretakers' misconception about vaccine contraindication, the result showed that 63(67.7%) of caretakers among cases and 69 (37.3 %) of caretakers among controls had misconception (Table 3).

**Table 2:** Health service utilization of care takers living in Lare district, Nuer Zone, Gambella, 2018

Variables	Categories	Cases N (%)	Controls N (%)
**Attend ANC**	Yes No	63(67.7) 30(32.3)	144(77.8) 41(22.2)
**Number of ANC**	1–2 3–4	44(69.8) 19(30.2)	70(48.6) 74(51.6)
**Place of delivery**	Home Health facility	55(59.1) 38(40.9)	92(49.7) 93(50.3)
**Attend PNC**	Yes No	23(24.7) 70(75.3)	100(54.1) 85(45.9)
**Missed opportunity**	Yes No	56(60.2) 37(39.8)	76(41.1) 109(58.9)
**Time taken to vaccination sites** **(in minute)**	≤30 >30	57(61.3) 36(38.7)	110(59.5) 75(40.5)

**Table 3:** Knowledge, perception and related factors of care takers living in Lare district, Nuer Zone, Gambela Region, 2018

Variables	Category	Cases N (%)	Controls N(%)
**Knowledge about**	Poor	68(73.1)	97(52.4)
**Vaccination**	Good	25(26.9)	88(47.6)
**Knowledge about schedule of**	Poor	59(63.4)	111(60)
**vaccination**	Good	34(36.6)	74(40)
**Knowledge about benefit of**	Poor	43(46.2)	65(35.1)
**vaccination**	Good	50(53.8)	120(64.9)
**Postponing vaccine schedule**	Yes	75(80.6)	85(45.9)
	No	18(19.4)	100(54.1)
**Heard about vaccine and VPD**	Yes	54(58.1)	143(77.3)
	No	39(41.9)	42(22.7)
**Retaining immunization card**	Yes	75(80.6)	141(76.2)
	No	18(19.4)	44(23.8)
**Misconception**	No misconception	30(32.3)	116(62.7)
	misconception	63(67.7)	69(37.3)
**Refusal of vaccination**	Yes	12(12.9)	15(8.1)
	No	81(87.1)	170(91.9)

The study found that 34(36.6%) of caretakers among cases and 137(74.1%) of caretakers among controls had been visited at least once per month while satisfaction of caretakers towards health workers was 34 (36.6%) among cases and 105 (56.8%) among controls.

The odds of incomplete childhood vaccinations were 2.16 [95% CI: 1.08–4.28] times more likely among caretakers of children who did not attend PNC at least once than caretakers who attended. The odds of incomplete childhood vaccinations were 6.15 [95% CI: 3.08–12.27] times more likely among caretakers who postpone vaccination schedule compared to caretakers who did not postponed their schedules. The odds of incomplete childhood vaccinations was 3.99 [95% CI: 2.13–7.48] times more likely among HH who had not been visited at least once per month by health workers than HH who had been visited. The odds of incomplete childhood vaccinations was 1.97 [95% CI: 1.04–3.74] times more likely among caretakers who did not satisfied with health workers compared to caretakers who were satisfied towards health workers. The odds of incomplete childhood vaccinations was 2.33 [95% CI: 1.19–4.59] times more likely among caretakers of children who had poor knowledge about vaccination than caretakers of children who knew about vaccination. The odds of incomplete childhood vaccinations was 2.90 [95% CI: 1.53–5.52] times more likely among caretakers who had misconception for vaccine contraindication than caretakers who did not have misconception.

In bivariate analysis 18 variables: age, educational status, mobility and marital status of caretakers, family size, birth order of the child, ANC attendance, PNC attendance, places of delivery, missed opportunity, knowledge about vaccination, the benefit of vaccination, postponing vaccine schedule, whether they heard about vaccination, misconception about the vaccine, refusal of vaccination, health workers' visit, and satisfaction of caretakers, were considered for further analysis with multiple logistic regression.

In multivariable analysis, PNC followup, postponing vaccination schedule, health worker visit, the satisfaction of caretakers, knowledge about vaccination and misconception for vaccination were found to be determinant of incomplete vaccination (Table 4).

**Table 4:** Multivariable logistic model for determinants of incomplete childhood vaccination in Lare district, Nuer zone, Gambela Region, 2018

Variables	Vaccination status		COR(95%CI)	AOR(95%CI)
			
	Cases N (%)	Controls N (%)		
**PNC follow up**				
**Yes**	23(24.7)	100(54.1)	1	1
**No**	70(75.3)	85(45.9)	3.58(2.06–6.22)	2.16(1.08–4.28)*
**Postponing vaccination** **schedule**				
**Yes**	75(80.6)	85(45.9)	4.90(2.72–8.84)	6.15(3.08–12.27)**
**No**	18(19.4)	100(54.1)	1	
**Health worker visit**				
**Yes**	34(36.6)	137(74.1)	1	1
**No**	59(63.4)	48(25.9)	4.95(2.90–8.46)	3.99(2.13–7.48)**
**Satisfaction** **of care takers**				
**Not satisfied**	59(63.4)	80(43.2)	2.27(1.36–3.80)	1.97(1.04–3.74)*
**Satisfied**	34(36.6)	105(56.8)	1	1
**Knowledge about** **vaccination**				
**Poor**	68(73.1)	97(52.4)	2.47(1.44–4.24)	2.33(1.19–4,59)*
**Good**	25(26.9)	88(47.6)	1	1
**Misconception for** **vaccination**				
**No misconception**	30(32.3)	116(62.7)	1	1
**Had misconception**	63(67.7)	69(37.8)	3.53(2.08–5.98)	2.90(1.53–5.52)**

** significant at p-value <0.001

* significant at p-value<0.05

1 reference group

## Discussion

This study identified determinants of incomplete childhood vaccination among children aged 12–23 months living in Lare district, Gambela region. The factors identified were postponing vaccination schedule by caretakers, household (HH) not visited by health workers, unsatisfied caretakers towards health workers, misconception about vaccination, poor knowledge about vaccination, and failure of caretakers to attend PNC followup.

Caretakers who did not attend PNC were associated with incomplete childhood vaccination. This finding supports the finding from Tigray and Machakel (14,15). In the current study, the proportion of health service utilization has decreased at the post-natal period in both cases and controls since the proportion of PNC follow-ups is less than the ANC counters. This might be due to maternal care interruption which brought differences in childhood vaccination status.

In this study, children from HH who were not visited at least once per month were more likely to have incomplete basic vaccinations than HH who were visited at least once per month. The finding was inline with study done in Tigray, Jigjiga and Kenya (15, 16 & 17). This implied that those HH who have been visited by healthcare providers might have got information about the time of their next schedules, and unvaccinated children could be easily traced and linked to a health facility. However, in contrast to this finding, the study done in Angola showed that health workers' visit had no significant association with completion of vaccination (18). This might be due to a difference in the way the health care providers interacted with the mothers/care takers suggesting that the mere visit of the health care providers only might not result in high quality performance of the heath care providers in improving childhood vaccination.

Analysis of grey literature on why children are not fully vaccinated showed that not only caretakers but also some health workers have misconception (false contraindication) for vaccinating a sick child with underweight, fever, respiratory diseases and diarrhoea which were reasons for postponing vaccination schedules and missing opportunity (19). This study also revealed that wrong belief (misconception) of caretakers for vaccination was found to be another factor associated with incomplete childhood vaccination. This finding was inline with study done in Machakel and Nepal (14, 20).

Postponing vaccination schedule was found to be associated with incomplete vaccination. This finding is in line with the studies done in Wonago, Yaoundé, Nepal and Malaysia (20,21,22,23). This might be due to factors related with child and caretakers, such as child sickness, sickness of caretakers, conflicting priorities, forgetting the schedule, or health-related factors such as time of delivery service, failure to inform caretakers about the need of timely vaccination of subsequent doses, long waiting time. In the study area, the main difference between cases and controls were forgetting the schedule and inconvenient time.

The finding of this study also revealed that unsatisfied caretakers towards skills, attitude and behaviors of health workers were associated with incomplete childhood vaccination. The finding of this study supported the evidence obtained by analysis of grey literature suggesting that skills, attitude and behaviors of health workers towards caretakers treating them unfriendly, disrespectfully, or even abusive manner towards caretakers might be resulted in discouraging children vaccination (19). This finding is also in line with the study done in Wonago and Kenya demonstrating that more satisfied caretakers might have positive perception towards health workers and more likely encouraged to seek vaccination thereby completing the recommended vaccines for their child. This might also imply that when the practice of health workers in a normal way with caretakers, there might be good communication so that caretakers can easily discuss with other caretakers (17,23). However, according to the EPI survey in 2015 report, more than a quarter of mothers were not communicated (24). In contrast to this finding, the satisfaction of caretakers was not associated with complete childhood vaccination in Machakel (14). This might be due to a difference in the attitude of caretakers towards health workers.

Poor knowledge about vaccination was found to be associated with incomplete childhood vaccination. This was in line with study conducted in Mizan. Good knowledge of the mothers/care takers also encourage mothers/caretakers to easily know whether the vaccination had been completed or not (25).

In the current study, there was no association between sociodemographic variables of caretakers with childhood vaccination status. This is consistent with the study done in Ambo and Mozambique (26,27). EPI survey which was done in selected zones of Ethiopia in 2015 also revealed that there was a variation between sociodemographic characteristics of caretakers with childhood vaccination status in different areas. This might be due to differences in exposure to information, differences in knowledge, attitude, and practice of caretakers, and variation in the strength of health service delivery (24).

The main limitation of this study was social desirability bias caused because of self-report of sociodemographic and caretakers perceptions. Facility level readiness for improving immunization delivery service was not also assessed.

In conclusion, mothers of children who did not attended PNC followup, postponing vaccination schedules, misconception for vaccine contraindication, knowledge about vaccine, unsatisfied caretakers towards health workers, and HH not visited by health workers were determinants of incomplete childhood vaccination.

Based on the finding, it is recommended that health education should be given for child caretakers about the importance of postnatal care and vaccinations. It is also recommended to improve health worker household visit and policy to strengthen the immunization program.

## References

[1] WHO (2015). immunization in the African region.

[2] (2014). REPORT OF THE SAGE WORKING GROUP ON VACCINE HESITANCY.

[3] Strengthening global immunization system.

[4] Federal Ministry of Health National EPI comprehensive multiyear plan 2016–2020.

[5] Central Statistical Agency (CSA) [Ethiopia] and ICF (2016). EDHS 2016.

[6] The Federal Democratic Republic of Ethiopia Ministry of Health HSTP Health Sector Transformation Plan.

[7] Borba RC, Vidal VM, Moreira LO (2015). The reemergency and persistence of vaccine preventable diseases. Anais da Academia Brasileira de Ciências.

[8] Salmon DA, Moulton LH, Omer SB, DeHart MP, Stokley S, Halsey NA (2005). Factors associated with refusal of childhood vaccines among parents of school-aged children: a case-control study. Archives of pediatrics & adolescent medicine.

[9] WHO (2017). 2017 Assessment Report of the GVAP SAGE on Immunization.

[10] Patel MK, Gacic-Dobo M, Strebel (2016). Progress Toward Regional Measles Elimination – Worldwide, 2000–2015. MMWR Morb Mortal Wkly Rep.

[11] Central Statistical Agency (CSA) [Ethiopia] and ICF (2016). EDHS 2016.

[12] SI research and training institute I (2015). EPI coverage in selected Ethiopian zones : A baseline survey for L10K's Routine Immunization Improvement Initiative.

[13] GRHB Gambela Regional RED database 2017 (1). unpublished report.

[14] Yenit MK, Assegid S, Abrha H (2015). Factors associated with incomplete childhood vaccination among children 12-23 months of age in Machakel Woreda, East Gojjam Zone: a Case-control Study. Journal of Pregnancy and Child Health.

[15] Aregawi HG, Gebrehiwot TG, Abebe YG, Meles KG, Wuneh AD (2017). Determinants of defaulting from completion of child immunization in Laelay Adiabo District, Tigray Region, Northern Ethiopia: A case-control study. PLoS One.

[16] Mohamud AN, Feleke A, Worku W, Kifle M, Sharma HR (2014). Immunization coverage of 12-23 months old children and associated factors in Jigjiga District, Somali National Regional State, Ethiopia. BMC Public Health.

[17] Kawakatsu Y, Honda S (2012). Individual-, family and community-level determinants of full vaccination coverage among children aged 12-23 months in western Kenya. Vaccine.

[18] Curry DW, Perry HB, Tirmizi SN, Goldstein AL, Lynch MC (2014). Assessing the effectiveness of house-to-house visits on routine oral polio immunization completion and tracking of defaulters. Journal of health, population, and nutrition.

[19] Favin M, Steinglass R, Fields R, Banerjee K, Sawhney M (2012). Why children are not vaccinated: a review of the grey literature. International health.

[20] Shrestha S, Shrestha M, Wagle RR, Bhandari G (2016). Predictors of incompletion of immunization among children residing in the slums of Kathmandu valley, Nepal: a case-control study. BMC public health.

[21] Ahmad NA, Jahis R, Kuay LK, Jamaluddin R, Aris T (2017). Primary immunization among children in malaysia: Reasons for incomplete vaccination. J Vaccines Vaccin.

[22] Félicitée ND, Christiane T, Roger D, Sandra T, Stève FW, Andreas C, Innocent K, Marie K (2016). Factors Influencing Routine Vaccination of Children of Mothers Live-Stock Retailers in the Markets of Yaoundé. World Journal of Vaccines.

[23] Tadesse H, Deribew A, Woldie M (2009). Predictors of defaulting from completion of child immunization in south Ethiopia, May 2008-A case control study. BMC public health.

[24] JSI research and training institute I (2015). EPI coverage in selected Ethiopian zones : A baseline survey for L10K's Routine Immunization Improvement Initiative [*Internet*].

[25] Meleko A, Geremew M, Birhanu F (2017). Assessment of child immunization coverage and associated factors with full vaccination among children aged 12-23 months at Mizan Aman town, bench Maji zone, Southwest Ethiopia. International journal of pediatrics.

[26] Jani JV, De Schacht C, Jani IV, Bjune G (2008). Risk factors for incomplete vaccination and missed opportunity for immunization in rural Mozambique. BMC public health.

[27] Etana B, Deressa W (2012). Factors associated with complete immunization coverage in children aged 12–23 months in Ambo Woreda, Central Ethiopia. BMC public health.

